# Resistance of Foxp3+ Regulatory T Cells to Nur77-Induced Apoptosis Promotes Allograft Survival

**DOI:** 10.1371/journal.pone.0002321

**Published:** 2008-05-28

**Authors:** Ran Tao, Wayne W. Hancock

**Affiliations:** Department of Pathology and Laboratory Medicine, Stokes Research Institute and Biesecker Pediatric Liver Center, Children's Hospital of Philadelphia and University of Pennsylvania, Philadelphia, Pennsylvania, United States of America; New York University School of Medicine, United States of America

## Abstract

The NR4A nuclear receptor family member Nur77 (NR4A1) promotes thymocyte apoptosis during negative selection of autoreactive thymocytes, but may also function in mature extrathymic T cells. We studied the effects of over-expression of Nur77 on the apoptosis of murine peripheral T cells, including thymic-derived Foxp3+ regulatory (Treg) cells. Overexpression of Nur77 in the T cell lineage decreased numbers of peripheral CD4 and CD8 T cells by ∼80% compared to wild-type (WT) mice. However, the proportions of Treg cells were markedly increased in the thymus (61% of CD4+Foxp3+ singly positive thymocytes vs. 8% in WT) and secondary lymphoid organs (40–50% of CD4+Foxp3+ T cells vs. 7–8% in WT) of Nur77 transgenic (Nur77Tg) mice, and immunoprecipitation studies showed Nur77 was associated with a recently identified HDAC7/Foxp3 transcriptional complex. Upon activation through the T cell receptor in vitro or in vivo, Nur77Tg T cells showed only marginally decreased proliferation but significantly increased apoptosis. Fully allogeneic cardiac grafts transplanted to Nur77Tg mice survived long-term with well-preserved structure, and recipient splenocytes showed markedly enhanced apoptosis and greatly reduced anti-donor recall responses. Allografts in Nur77Tg recipients had significantly increased expression of multiple Treg-associated genes, including Foxp3, Foxp1, Tip60 and HDAC9. Allograft rejection was restored by CD25 monoclonal antibody therapy, indicating that allograft acceptance was dependent upon Treg function in Nur77Tg recipients. These data show that compared to conventional CD4 and CD8 T cells, Foxp3+ Tregs are relatively resistant to Nur77-mediated apoptosis, and that tipping the balance between the numbers of Tregs and responder T cells in the early period post-transplantation can determine the fate of the allograft. Hence, induced expression of Nur77 might be a novel means to achieve long-term allograft survival.

## Introduction

Nur77 (also known as NR4A1, TR3, NGFI-B or NAK1) is an inducible orphan nuclear receptor comprised of an N-terminal AF1 transactivation domain, a DNA binding domain containing two zinc fingers, and a C-terminal ligand binding domain. Nur77 is necessary for induction of apoptosis in T-cell hybridomas [1], [2] and constitutive expression of Nur77 in thymocytes and various cell lines leads to apoptosis [3], [4]. In vivo, transgenic mice expressing dominant-negative Nur77 are defective in the process of negative selection whereas positive selection proceeds normally [3], [5]. However, Nur77-deficient mice are phenotypically normal, suggesting functional redundancy between Nur77 and related proteins [6].

Aspects of the mechanism by which Nur77 induces cell apoptosis remain unclear. In resting thymocytes, Nur77 expression is inhibited by the histone deacetylase, HDAC7 [7]. T cell receptor (TCR) activation induces HDAC7 phosphorylation by the serine/threonine kinase PKD1, and phosphorylated HDAC7 binds to 14-3-3 protein and is exported from the nucleus, leading to depression of Nur77 and other gene targets [7]–[10]. However, Nur77 downstream target genes responsible for regulating T cell apoptosis remain to be identified. Although expression levels of FasL and CD30 are elevated in Nur77 transgenic mice [4], Nur77-mediated apoptosis is still intact in gld/gld (FasL mutant mice) or CD30−/− mice, precluding FasL and CD30 as the major downstream effectors of Nur77-related apoptosis in thymocytes [11]. Moreover, negative selection of CD4+CD8+ doubly-positive thymocytes is intact in Fas- or CD30-deficient mice, and constitutive expression of Nur77 in gld/gld mice rescues the lymphoproliferative phenotype of the FasL mutant mice [12], [13]. In tumor cell lines, Nur77 initiates apoptosis by translocating to the mitochondria, leading to release of cytochrome c [14], [15], activation of Apaf-1 and caspase-9, cleavage of pro-caspase 3 and induction of apoptosis [16]. Over-expression of Bcl-2 does not block Nur77-mediated apoptosis in T cells [11], suggesting that either Nur77 transcription plays an especially prominent role in initiating apoptosis in T cells or that Nur77 initiates a Bcl-2-independent mitochondrial apoptotic pathway. Indeed, recent microarray studies showed Nur77 induces known apoptotic genes such as FasL and TRAIL, but also novel genes such as NDG1 and NDG2 that promote apoptosis in a Bcl-2-independent manner [17].

In addition to promoting cell apoptosis through the mechanisms outlined above, Nur77 can also function as an inhibitor of protein kinase C (PKC) [18]. The C-terminal ligand-binding domain of Nur77 can specifically interact with the highly conserved glycine-rich loop of PKC-theta that is required for its catalytic activity, preventing the phosphorylation of substrate by PKC-theta. Hence, inhibition of catalytic activity by Nur77 can repress PKC-mediated activation of AP-1 and NF-κB, with multiple downstream effects including preventing induction of anti-apoptotic genes.

All these findings suggest an essential role of Nur77 in apoptotic death accompanying negative selection in thymocyte development. However, the role of Nur77 in induction of apoptosis in mature T cells, and in the induction and/or maintenance of peripheral tolerance is only poorly understood. Likewise, there are no data as to the effects of Nur77 on the apoptosis of thymic-derived Foxp3+ regulatory T (Treg) Treg vs. non-Treg cells. In the current study, we evaluated the role of Nur77 on peripheral T cell apoptosis by taking advantage of transgenic mice in which Nur77 was overexpressed in thymocytes and T cells using the Lck promoter.

## Results

### Effect of Nur77 over-expression on peripheral T cell populations

**Figure 1:**
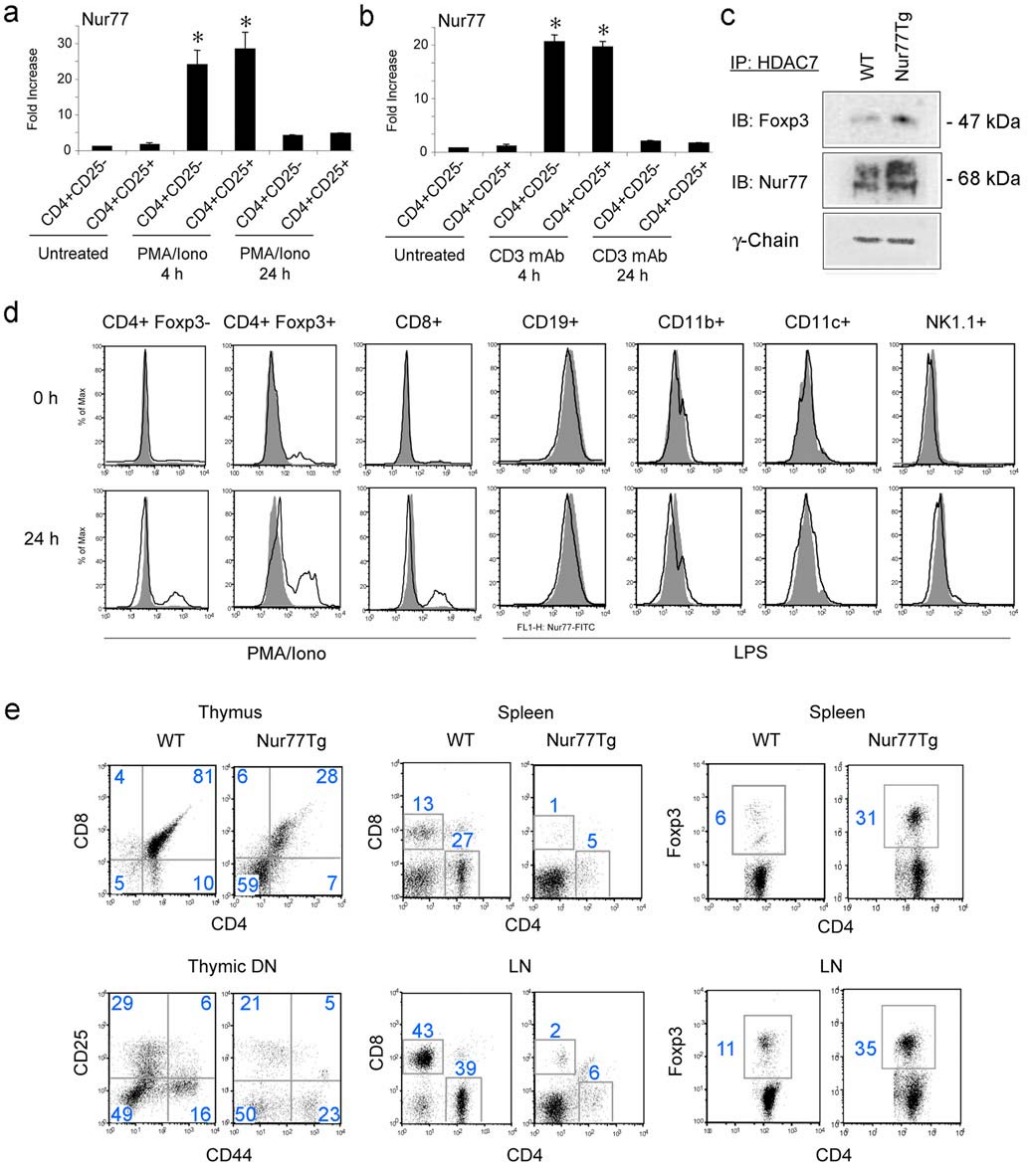
Nur77 expression by thymocytes and peripheral T cells in WT and Nur77Tg mice. (a) Nur77 mRNA expression after T cell activation using PMA/ionomycin or (b) CD3 mAb as shown by qPCR analysis of CD4+CD25− and CD4+CD25+ T cells; data expressed as fold increases (mean±SD) above control set as 1. Nur77 expression at 4 h was comparable (p>0.05) for Tregs and non-Tregs, but significantly increased compared to levels either at rest or after 24 h of activation (*p<0.01). (c) Nur77 forms a complex with HDAC7 and Foxp3 as shown by immunoprecipitation of HDAC7 from splenocytes, followed by SDS-PAGE and Western blotting with mAb to Foxp3 or Nur77. (d) Nur77 is mainly expressed by T cells upon activation, especially by CD4+Foxp3+ Tregs, as shown by splenocytes stimulated with PMA/ionomycin or LPS for 24 h, followed by staining with cell markers, and mAbs to Foxp3 and Nur77, or isotype controls (grey). Data are representative of 2 experiments with similar results. (e) Altered thymocyte development, decreased peripheral T cell populations and expanded proportion of CD4+Foxp3+ fraction in Nur77Tg mice. Thymuses from WT or Nurr77Tg mice were stained with CD4, CD8, CD25 and CD44 Abs; left upper panel showed the proportions of single positive (CD4+ or CD8+), double positive and double negative cells, and left lower panel shows analysis of the double negative (DN) population 1–4 based on CD44 and CD25 expression. Cells were also isolated from spleens and lymph nodes of WT or Nur77Tg mice and stained with CD4 and CD8 Abs, followed by intranuclear staining for Foxp3. Figure in each square indicates the percentage of labeled cells, and data are representative of results from 8 mice per group.

We found by quantitative real-time PCR (qPCR), that expression of the inducible immediate early gene, Nur77, was upregulated within 4 h of *in vitro* stimulation by PMA/ionomycin (Figure 1a) or CD3 monoclonal antibody (mAb) (Figure 1b), and returned to basal levels by 24 h in both non-Tregs and Tregs (p>0.05). Using immunoprecipitation, we found that Nur77 formed a complex with HDAC7 and Foxp3 in resting Tregs (Figure 1c), similarly to that of HDAC7 and Foxp3 [19], and intranuclear staining for Nur77 protein showed that Nur77 was expressed by a small population of naive CD4+ T cells, especially CD4+ Foxp3+ T cells, but not by naive CD8+ T cells (Figure 1d). After 24 h of in vitro stimulation, both CD4+ and CD8+ T cells showed increased Nur77 protein expression, though levels of expression were less than in CD4+ Foxp3+ T cells. Nur77 was not expressed by CD19+ B cells, CD11b+ macrophages, CD11c+ dendritic cells or NK1.1+ natural killer cells, regardless of LPS stimulation (Figure 1d).

In agreement with a previous analysis of these mice [3], we found that thymocytes in Nur77Tg mice were markedly expanded at the double-negative stage (DN, 59% vs. 5% in WT mice), with little change in distribution of cells within DN1–DN4 (Figure 1e). However, both CD4 and CD8 T cell populations in Nur77Tg mice were significantly decreased in the spleen (5% CD4+ T cells vs. 27% in WT mice, 1% CD8+ T cells vs. 13% in WT mice) and lymph nodes (6% CD4+ T cells vs. 39% in WT mice; 2% CD8+ T cells vs. 43% in WT mice) (Figure 1e). There was also a marked expansion of the CD4+ Foxp3+ T cell population in Nur77Tg mice compared to WT controls (Figure 1e), with increases in the Foxp3+ to Foxp3− ratio of the spleen (1∶2 vs. 1∶15 in WT mice) and lymph nodes (1∶2 vs. 1∶9 in WT mice). Thus, Nur77Tg mice were not only lymphopenic but possessed an expanded population of Tregs in their peripheral lymphoid organs.

### Activation-induced apoptosis of Nur77Tg CD4 and CD8 but not Treg cells

**Figure 2:**
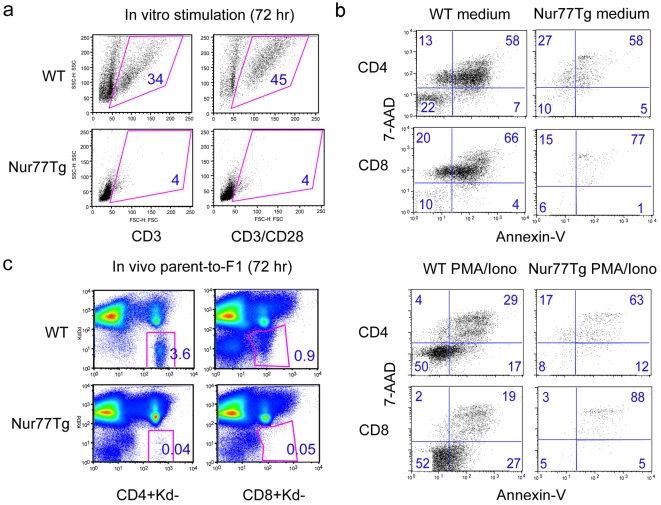
Increased apoptosis of Nur77Tg T cells upon activation in vitro and in vivo. Splenocytes from WT or Nur77Tg mice were stimulated with PMA/ionomycin for 72 h, followed by flow cytometry using T cell markers and Annexin-V and 7-AAD staining. (a) Live cells were gated based on forward vs. side scatter, and (b) T cell apoptosis was based on Annexin-V and 7-AAD staining. (c) CFSE-labeled B6 or Nur77Tg spleen and lymph node cells were adoptively transferred into B6/DBA F1 mice. After 72 h, donor-derived live CD4+ or CD8+ T cells were identified by gating on H-2K^d^ (-) and H-2D^d^ (-) cells; figure in each square indicates percentage of the gated population. Data are representative of 3 experiments with similar results.

Although Nur77 over-expression causes increased thymocyte apoptosis during development, its effects on peripheral T cell apoptosis after activation are unclear. Whole splenocytes from WT or Nur77Tg mice were stimulated for 72 h with soluble CD3 mAb (1 µg/ml) alone or CD3 plus CD28 mAbs (0.5 µg/ml). Far fewer live Nur77Tg cells were recovered after in vitro stimulation as compared to WT splenocytes (Figure 2a). Similar differences were seen after stimulation for 72 h with PMA plus ionomycin; both CD4+ and CD8+ T cells from Nur77Tg mice showed significantly increased apoptosis after activation as compared to WT T cells (Figure 2b). We used a parent-to-F1 adoptive transfer model to assess the effects of Nur77 over-expression on T cell survival upon allogeneic stimulation in vivo. Use of Nur77Tg mice as a source of donor T cells led to a markedly reduced recovery of T cells after 72 h as compared to use of T cells from WT mice (Figure 2c), suggesting markedly increased death of Nur77Tg T cells as a result of alloantigen-induced T cell activation and proliferation in vivo.

**Figure 3:**
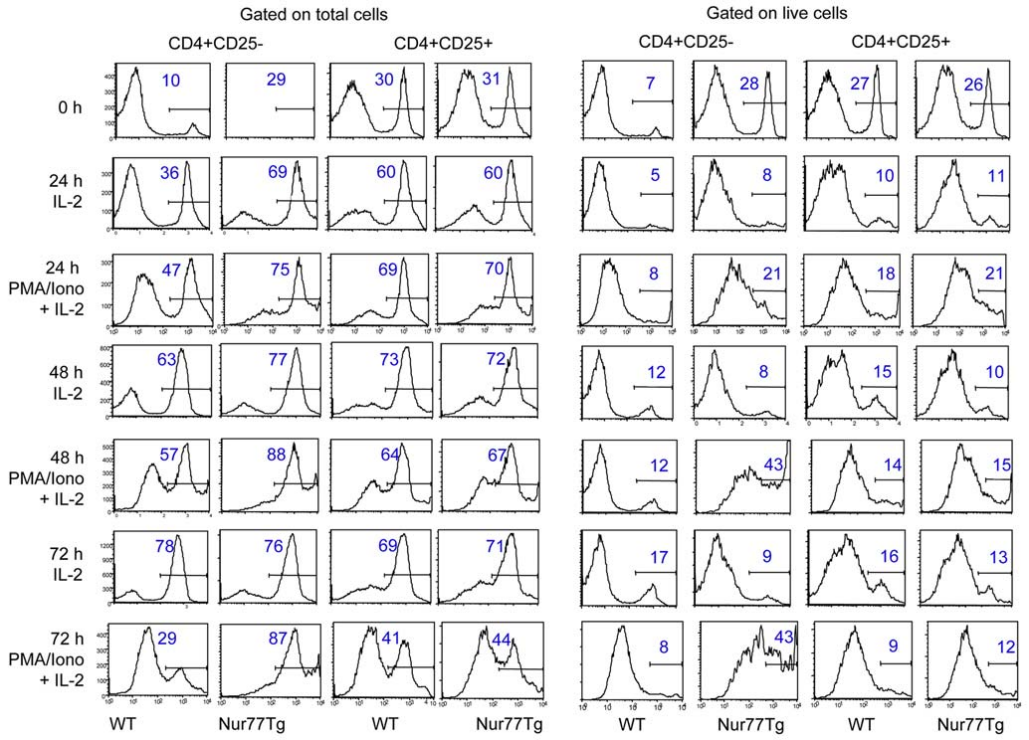
Resistance of Treg cells to activation-induced apoptosis. CD4+CD25− and CD4+CD25+ were isolated by MACS separation and stimulated in vitro with PMA/ionomycin plus IL-2 for 0, 24, 48 and 72 h (IL-2 alone as no stimulation control). T cell apoptosis at the indicated time points was detected by Annexin V staining and flow cytometry analysis. Histogram data with the figure in each gated population indicating percentage of Annexin V+ cells in the total (left panels) or live (right panels) cell populations. Data are representative of 3 experiments with similar results.

To directly compare the impact of Nur77 over-expression on susceptibility to apoptosis in non-Treg vs. Treg cells, CD4+CD25− and CD4+CD25+ T cell populations were purified by MACS separation and stimulated in vitro with PMA/ionomycin and IL-2; T cell apoptosis was detected by Annexin V staining. Nur77 overexpression led to increased apoptosis of untreated non-Treg cells compared to WT cells, whereas Tregs from both Nur77Tg and WT mice showed comparable low rates of apoptosis at serial time points (Figure 3). Assessment of pro-apoptotic and anti-apoptotic gene expression by resting and activated cells showed comparable data for Nur77Tg Treg cells and non-Treg cells except that Nur77Tg Tregs showed greater upregulation of Bcl-2 (p<0.01) upon activation (Figure S1). Collectively, these data indicate that Treg cells are markedly more resistant to Nur77-induced apoptosis than non-Treg cells.

### Impaired proliferation of Nur77Tg T cells after activation

**Figure 4:**
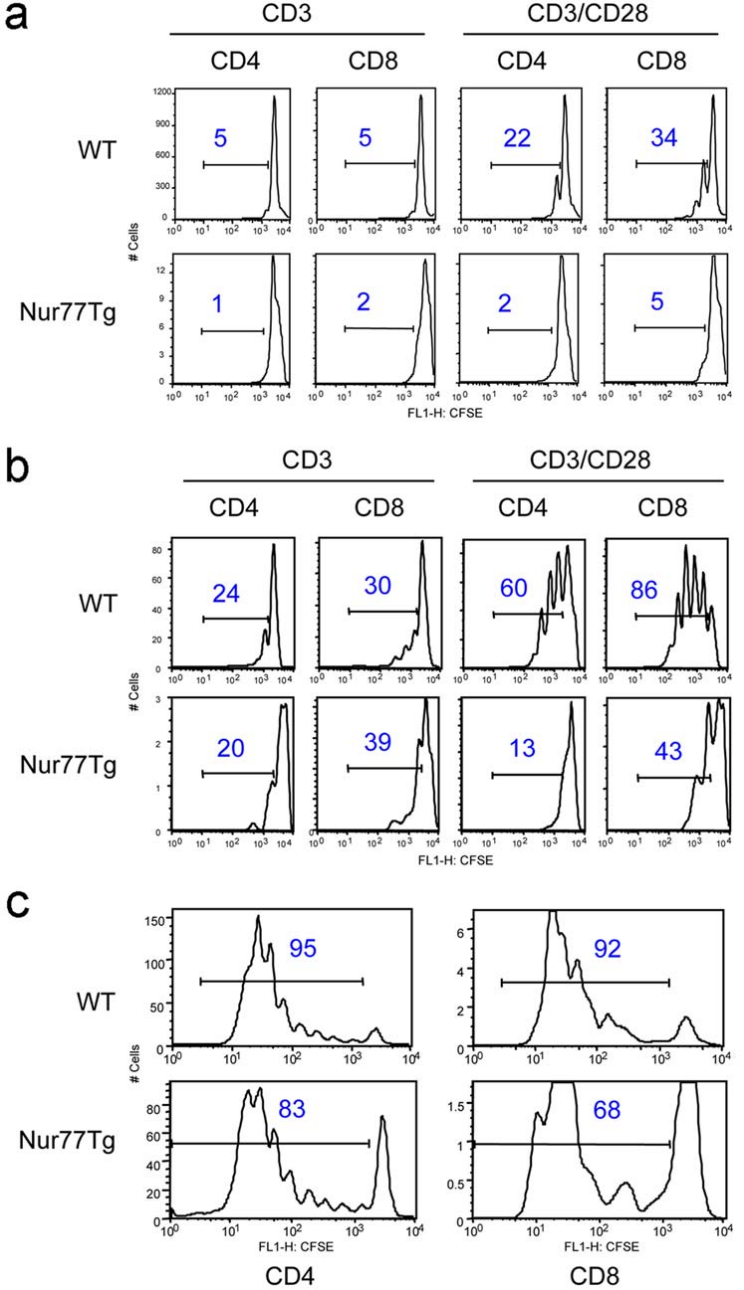
Impaired proliferation of Nur77Tg T cells. (a) Impaired T cell proliferation of CFSE-labeled Nur77Tg mice after 48 h of in vitro activation with soluble CD3 mAb alone or plus CD28 mAb; figure in each square indicates percentage of the gated population. (b) Assessment of T cell proliferation after 72 h; experimental details as for previous panel. (c) Impaired proliferation of Nur77Tg T cells after allo-stimulation in vivo. Allogeneic T cell responses were generated by i.v. injection of CFSE-labeled B6 or Nur77Tg spleen and lymph node cells into B6/DBA F1 recipients. !After 72 h, donor derived allo-reactive T cells were identified by gating on H-2K^d^ (-) and H-2D^d^ (-) cells and T cell proliferation assessed by CFSE division profiles; note different y-axis for WT vs. Nur77Tg T cells. Figure in each square indicates percentage of the gated population; data representative of 3 experiments with similar results.

To assess the effects of Nur77 on T cell activation and proliferation, WT or Nur77Tg splenocytes were labeled with CFSE and stimulated with soluble CD3 or CD3 plus CD28 mAbs for 48 to 72 h. T cells from Nur77Tg mice showed much more apoptosis than T cells from WT mice after in vitro activation, but residual T cell proliferation was detected after 48 h (Figure 4a) or 72 h (Figure 4b) of TCR ligation with or without CD28 costimulation, albeit to a less extent than WT controls. Similar results were seen in vivo, using a parent-to-F1 adoptive transfer model in which only the transferred donor T cells proliferate. Nur77Tg T cells were still capable of proliferation upon allo-activation in vivo, but with decreased proliferation capacity compared to WT, and with lesser T cell recovery (Figure 4c). These results show that Nur77Tg T cells proliferate less than WT T cells in vitro and in vivo, consistent with compromise of T cell proliferative responses by Nur77-induced apoptosis of activated T cells.

### Long-term allograft acceptance in Nur77Tg recipients

**Figure 5:**
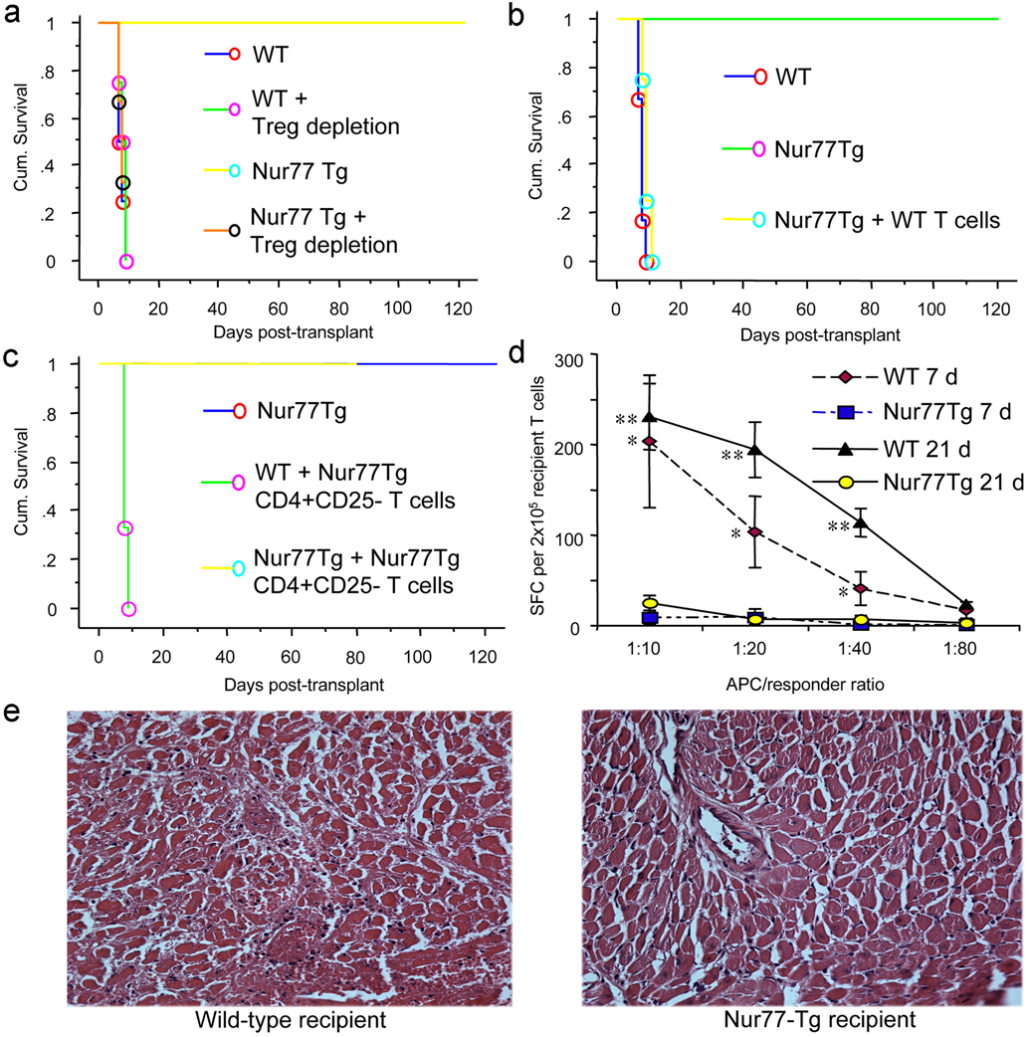
Long-term acceptance of MHC mismatched cardiac allograft in Nur77Tg recipients. (a) BALB/c cardiac allografts were accepted long-term by Nur77Tg recipients, in contrast to acute rejection in WT C57BL/6 recipients (p<0.001). Long-term survival was dependent upon Treg cell function since Treg cell depletion by thymectomy and CD25 mAb therapy pre-transplant restored acute rejection in Nur77Tg recipients (p<0.001 vs. control Nur77Tg mice). (b) Adoptive transfer of WT T cells restored acute allograft rejection in Nur77Tg mice (p<0.01); 2×10^6^ CD4+CD25− WT T cells purified using magnetic beads were injected i.v. injected into Nur77Tg recipients (n = 4) immediately post-transplant. (c) Adoptive transfer of additional Nur77Tg T cells did not restore allograft rejection in Nur77Tg recipients; 1×10^7^ CD4+CD25− WT T cells purified using magnetic beads were injected i.v. into Nur77Tg recipients (n = 4) immediately post-transplant. (d) Significantly decreased donor-specific responder frequency in Nur77Tg recipients of BALB/c cardiac allografts. Recipient spleens (n = 3/group) were harvested at 7 or 21 d post-transplant and CD3+ T cells were isolated by MACS separation; purified recipient T cells were re-stimulated in 96-well ELISPOT plates using irradiated donor splenocytes (donor-specific antigen) or recipient splenocytes (syngeneic controls) at different stimulator to responder ratios. Recipient anti-donor responder frequencies were determined by IFN-γ spot-forming cells (SFC)/2×10^5^ recipient T cells (mean±SD); *p<0.01 and ** p<0.005 for WT vs. Nur77Tg data at corresponding time post-transplant. (e) Acute rejection of BALB/c allografts in WT recipients (left) versus minimal leukocyte infiltration and myocardial or vascular injury in allografts harvested at 7 d post-transplant from Nur77Tg recipients (H&E-stained paraffin sections, original magnifications ×250).

We undertook cardiac allografting across a full major histocompatibility complex (MHC) disparity to assess the practical effects of Nur77 overexpression in a disease model. We found that whereas WT C57BL/6 (B6) mice rejected BALB/c donor heart with a mean survival time (MST) of 7.6±0.8 d (n = 6), Nur77Tg mice spontaneously accepted BALB/c cardiac grafts (MST>100 d, n = 6) (Figure 5a, p<0.001). Since Nur77Tg mice are lymphocytopenic but also contain a large fraction of CD4+CD25+ or CD4+Foxp3+ Treg cells, long-term allograft survival might reflect a lack of sufficient numbers of effector T cells to mount an alloresponse, their death by activation-induced apoptosis, or suppression of alloresponses by a disproportionately increased number of Foxp3+ Treg cells. To test these hypotheses, we first undertook thymectomy and treatment of Nur77Tg or WT mice with a depleting CD25 mAb (PC61) 7 days before cardiac transplantation; the efficacy of CD4+CD25+ T cell depletion was monitored by flow cytometry, as described [20]. Treg depletion in WT recipients did not affect the tempo of allograft rejection, but readily restored acute rejection of cardiac allografts in Nur77Tg recipients (Figure 5a, p<0.001), indicating the residual small populations of effector T cells in Nur77Tg mice could still mount an effective alloresponse in the absence of Tregs.

We next considered whether Nur77-induced apoptosis played a role in this transplant model. We evaluated secondary lymphoid organs of Nur77Tg recipients at 7 and 21 d post-transplant. Compared to WT recipients, there were markedly decreased CD4 and CD8 T cell populations in Nur77Tg recipients. Most of the CD4+ and CD8+ T cells in the spleen (Figure S2a) and lymph nodes (Figure S2b) of Nur77tg recipients were Annexin V-positive, indicating significantly enhanced apoptosis of Nur77Tg effector T cells upon allogeneic immune activation. By contrast, the lymphoid tissues of Nur77Tg recipients contained about two-fold more Foxp3+ Tregs as compared to the percentages of Tregs in lymphoid tissues of WT recipients (Figure S2c).

We undertook adoptive transfer studies to further assess whether Nur77-induced apoptosis was critical to permanent graft survival. As few as 2×10^6^ purified WT T cells injected intravenously into Nur77Tg recipients immediately after cardiac transplantation were able to restore acute rejection (Figure 5b, p<0.001). To test whether spontaneous allograft acceptance in Nur77Tg recipient was due to the high Treg:Teff cell ratio compared to WT mice, we adoptively transferred 10^7^ purified Nur77Tg CD4+CD25− T cells into Nur77Tg recipients to increase the size of the non-Treg population and restore the normal Treg:Teff ratio. Increasing the size of the Teff cell population in Nur77Tg recipients did not restore acute allograft rejection and allografts were maintained long-term (n = 4, p>0.05 compared to Nur77Tg recipients without adoptive transfer, Figure 5c). These data indicate that the enhanced rate of activation-induced apoptosis in Nur77Tg T cells renders them more susceptible to control by Nur77-resistant Tregs.

We assessed recall responses of memory T cells in Nur77Tg mice by ELISPOT assay. Since spleens of Nur77Tf mice were far less cellular than those of WT mice, CD3+ T cells were purified from WT and Nur77Tg recipient spleen 7 and 21 d post-transplant and re-stimulated in vitro with donor-derived allogeneic antigen-presenting cells (APC) or syngeneic controls (data not shown). In comparison to WT, Nur77Tg recipients had a significantly reduced donor specific responder frequency, as assessed by IFN-γ spot-forming cell numbers (Figure 5d). Collectively, these studies indicate that spontaneous allograft acceptance in Nu77Tg recipients results from both increased Treg cell numbers and increased activation-induced apoptosis of T effector cells.

### Intragraft gene expression

**Figure 6:**
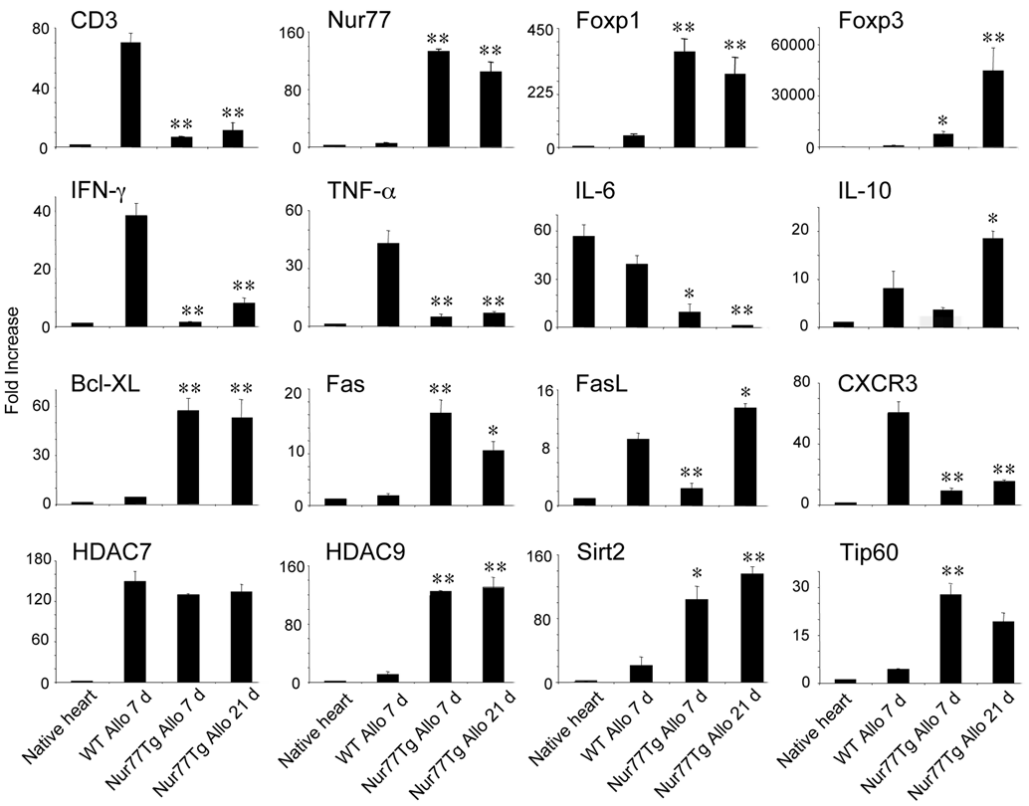
Intragraft gene expression in Nur77Tg versus WT recipients. (a) Total RNA was extracted from 7 d cardiac allografts of WT recipient, and 7 d and 21 d allografts of Nur77Tg recipients, and intragraft gene expression of Nur77, CD3, CD45, IL-6, TNF-α, Foxp1, Foxp3, GITR, CTLA4 and HDAC9 was assessed by q PCR. Data were expressed as fold increases (mean±SD) above the normal BALB/c heart; *p<0.05 and **p<0.01 versus corresponding WT allograft data. Data are representative of 3 experiments with similar results.

In contrast to cardiac allografts of WT mice harvested at day 7 post-transplant, histological examination showed only minor mononuclear cell infiltration, well-preserved myocardium and vasculature in allografts harvested from Nur77Tg recipients (Figure 5e). The latter grafts also had negligible numbers of TUNEL+ cells (data not shown), indicating that T cells underwent activation-induced apoptosis in secondary lymphoid organs rather than in the graft itself. We next undertook evaluation of intragraft gene expression by qPCR, grafts harvested at 7 d post-transplant from the two groups, as well as at 21 d post-transplant in the case of Nur77Tg recipients. Comparison of intragraft events at 7 d post-transplant showed that allografts from Nur77Tg had decreased levels of CD3 but markedly increased levels of Nur77, consistent with infiltration by small numbers of T cells that highly expressed Nur77 when compared to WT recipients (Figure 6). Nur77Tg allografts also contained increased levels of Treg-associated genes such as Foxp3 and HDAC9 [21] but only low levels of the cytokines IL-6, IFN-γ and TNF-α, suggesting significantly diminished inflammation and increased Treg recruitment into the allograft.

### Di-n-butylin dichloride induced Nur77 expression and apoptosis in T cells

**Figure 7:**
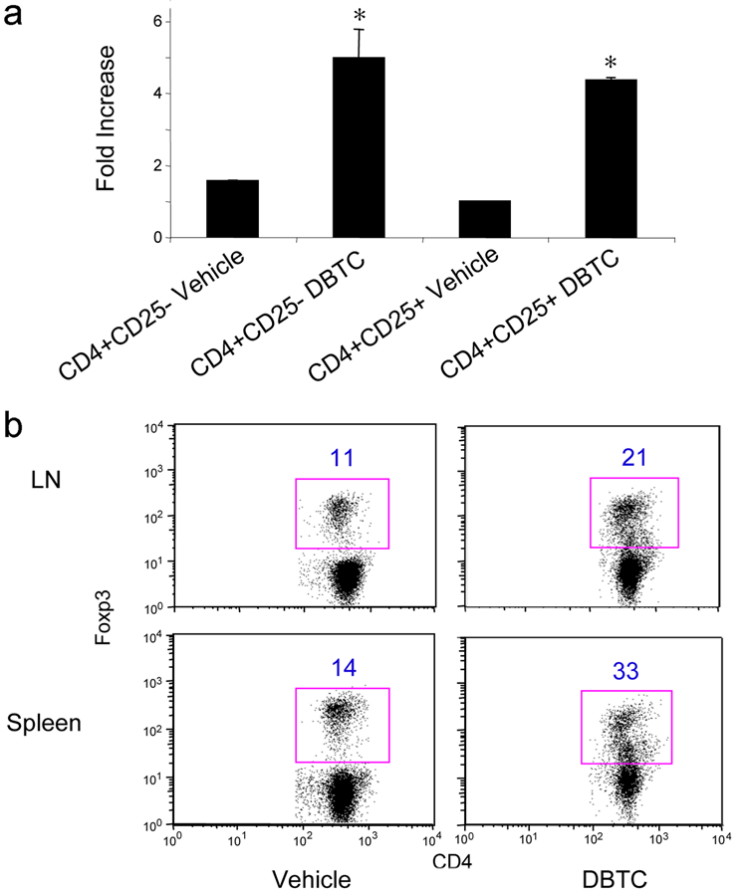
DBTC induces Nur77 and differentially promotes non-Treg vs. Treg cell apoptosis. (a) WT CD4+CD25− and CD4+CD25+ T cell populations were fractionated using magnetic beads (>90% purity) and cultured for 4 h in RPMI medium plus 3 µM DBTC or ethanol alone (final concentration of 0.1% ethanol). Nur77 mRNA expression was detected by qPCR; data are expressed as fold increase (mean±SD) above control set as 1. DBTC treatment increased Nur77 mRNA in both cell populations (p<0.05 for CD4+CD25− vs. control, p<0.01 for CD4+CD25 vs. control), but levels of induction at 4 h were not significantly different (p>0.05) for the groups treated with DBTC. (b) C57BL/6 mice were fed DBTC (60 mg/kg/day) for 3 d, sacrificed and single cell suspensions from spleens and lymph nodes were stained with T cell markers and Foxp3 mAb. Flow cytometric analysis of Foxp3 expression in the CD4+ T cell population is representative of 4 mice/group, and the percentage of the gated population is indicated.

Nur77 was identified by DNA microarray as an immediate early gene induced by a thymotoxic organotin compound, di-*n*-butylin dichloride (DBTC) [22]. While DBTC treatment in vitro and in vivo was shown to induce thymocyte apoptosis, we investigated whether DBTC had the same effect on peripheral T cells. Accordingly, WT CD4+CD25− and CD4+CD25+ T cell populations were fractionated using magnetic beads to >90% purity, and 1×10^6^ cells of each fraction were cultured in the presence of DBTC (3 µM, dissolved in ethanol) or ethanol carrier alone (the final concentration of ethanol in the culture medium was 0.1%). Cells were harvested after 4 h and Nur77 mRNA expression detected by qPCR. We found that DBTC treatment induced Nur77 expression equally in both CD4+CD25− and CD4+CD25+ T cell populations (p<0.05 compared to ethanol alone, Figure 7a). We also studied the effect of inducing T cell apoptosis in vivo with DBTC treatment. Mice were fed DBTC and apoptosis of non-Treg and Treg cells in vivo assayed by Annexin V staining after 72 h. DBTC treatment in vivo caused a markedly increase in the Treg vs. Teff ratio (Figure 7b), consistent with the differential effects of Nur77 transgene expression on CD4+CD25− T cells versus CD4+CD25+ Treg cells.

## Discussion

This study showed remarkable effects on the immune system caused by the over-expression of Nur77 in T cells. In the only previous study of these mice in a disease model, the incidence and severity of collagen-induced arthritis was significantly decreased in Nur77-transgenic mice versus WT controls [23]. Attenuation of the disease was associated with apoptosis of transgenic T cells and decreased production of collagen type II-specific IgG2a antibodies, consistent with impaired capacity for normal T cell proliferation and provision of help for T cell-dependent B cell functions. In contrast, we now show significant therapeutic consequences of the differential susceptibility of Treg versus non-Treg cells to undergo Nur77-induced apoptosis.

As expected, given its pro-apoptotic role during thymocyte development, Nur77 over-expression led to a marked reduction in the numbers of mature T cells in the periphery. However, the residual peripheral T cells themselves showed markedly increased rates of apoptosis upon activation in vitro or in vivo, indicating that Nur77 is an important regulator of mature T cell apoptosis. Despite their decreased numbers and increased rates of apoptosis following cell activation, in the absence of Tregs, T cells of Nur77Tg mice were able to promote rejection of allografts at a similar pace to that exerted by WT T cells. By contrast, CD4+CD25+ naturally occurring Tregs were more resistant to apoptosis induced by Nur77 over-expression, such that secondary lymphoid tissues of Nur77Tg mice had a markedly expanded population of Foxp3+ Tregs. The spontaneous long-term acceptance of fully MHC-mismatched cardiac allografts in this strong-responder strain combination highlights the extent to which the Treg to T effector cell ratio can dampen host immune responses.

Allografts in Nur77Tg recipients at both peri-transplant and later periods were infiltrated by Foxp3+ Tregs, and had increased expression of several genes recently implicated in Treg biology, including HDAC9, TIP60 and Foxp1 [19], [21]. They also showed decreased levels, compared to allografts in WT recipients, of pro-inflammatory cytokines and chemokine receptors important to allograft rejection in this model [24]. The finding that Nur77 could be co-immunoprecipitated with HDAC7, extends to Foxp3+ Tregs the known relationship of these two molecules in T cell development, especially within the thymus [3], [7]. Further characterization of the roles of HDAC7, HDAC9 and related proteins in Tregs is underway in our laboratory [19], [21].

Despite the presence of relatively large numbers of Tregs in Nur77Tg mice, adoptive transfer of small numbers of WT T effector cells led to prompt rejection of cardiac allografts, while transferring even large numbers of Nur77Tg T effector cells, so as to increase the T effector to Treg cell ratio, did not abrogate spontaneous allograft acceptance, pointing to the important role of T effector cell apoptosis in achieving transplant tolerance. Hence, efforts to induce allograft acceptance as a result of Nur77 over-expression will likely require enhanced expression in both CD4 and CD8 T cells whereas, at the least, associated enhancement of Nur77 expression in Treg cells does not appear to have negative consequences.

Previous studies showed using Foxp3 transgenic mice that increased expression of Foxp3 led to increased apoptosis of CD4+CD25− T cells following TCR activation, along with upregulation of pro-apoptotic genes such as FasL, TRAIL. TNF-α and Bad [25]. The Fas-FasL pathway was also implicated in the control of Treg apoptosis in studies of FasL mutant (gld) mice; these contain markedly increased numbers of CD4+CD25+ Tregs within the CD4 population [26]. Recent data suggest that adult human Foxp3+ Tregs are comprised of a naive (CD4+CD25+CD45RA^hi^) CD95− subset, as well as a more frequent memory (CD4+CD25+CD45RO) CD95+ subset, and that these two subsets differ in their susceptibility to CD95L-induced apoptosis [27]. Thus, while the memory subset is prone to CD95L-mediated death, naive Tregs are resistant. Tregs are known to be more resistant than conventional T cells to activation-induced cell death [28], and the current data indicate that Tregs are also more resistant than conventional Tregs to Nur77-induced cell death.

In addition to transgenic approaches, Nur77 expression can be modulated by physiologic and chemical agents. Nur77 was first identified as an immediate-early gene induced by serum (9) or TCR signaling in T cell hybridomas and thymocytes [1], [2]. Natural and synthetic retinoic acids affect Nur77 activity through its interaction with RAR and RXR; all-trans or 9-cis-retinoic acids, which block activation-induced cell death, inhibit the transcriptional activity of Nur77 (and expression of FasL) in T cells [29], [30]. By contrast, CD437, a RARγ-specific agonist, induces Nur77 expression through an unknown mechanism and enhances Nur77-mediated activity through a Nur77-binding response element [30], [31].

 With regard to effects of pharmacologic agents on Nur77 expression and function, thapsigargin inhibits endoplasmic reticulum-dependent Ca^2+^-ATPase, increases cytosolic Ca^2+^ levels, and induces Nur77 expression and causes apoptosis in T cells [32]. Likewise, Nur77 transcription triggered by TCR signaling can be inhibited by cyclosporin A, suggesting that calcineurin plays an important role in its regulation. Cyclosporin A blocks apoptosis by inhibiting the DNA binding activity of the transcription factor Nur77 through the N-terminal region of the protein [33]. Lastly, Nur77 was shown to be rapidly induced in vitro and in vivo in thymocytes exposed to DBTC, and antisense oligonucleotide inhibition of Nur77 expression prevented apoptosis induced by DBTC, suggesting that DBTC caused thymocyte apoptosis by inducing Nur77 expression [22].

Given our initial data, DBTC may be a suitable agent to test the effects of pharmacologic induction of Nur77 in T cells, including in experimental allograft models. Future studies from our group will involve testing of this compound in allograft models, as well as assessment of the effects of engineering of a dominant-negative form of Nur77 on T cell and Treg function. Collectively, the current data, along with the proposed studies, should provide a basis for assessing whether manipulation of Nur77 levels by pharmacologic or genetic means warrants further development and testing in pre-clinical disease models.

## Materials and Methods

### Mice

Mice transgenic for Nur77, under control of the T cell–specific lck proximal promoter as described [3], were backcrossed to the C57BL/6 strain and maintained in our animal facility. WT C57BL/6 (H-2^b^), BALB/c (H-2^d^), B6D2F1 and congenic Thy1.1 mice were purchased from The Jackson Laboratory (Bar Harbor, ME). All studies were performed with a protocol approved by the institutional animal care and use committee of The Children's Hospital of Philadelphia.

### Reagents

We purchased CFSE (Molecular Probes), DBTC (Sigma), antibodies against CD25 (PC-61, eBioscience), Foxp3 (Alexis), HSC70 (Santa-Cruz), HDAC7 (Sigma), HDAC9 (BioVision), Nur77 (IMG-528, IMGENEX), mAbs to cell lineage markers (BD-PharMingen), and HRP-conjugated anti-rabbit, rat or goat IgG antibodies (Santa-Cruz) for immunoprecipitation, Western blotting and intranuclear staining.

### Flow cytometry

Allogeneic T cell responses were generated by i.v. injection of 30×10^6^ CFSE-labeled B6 spleen and lymph node cells into B6/DBA F1 recipients, a parent→F1 MHC mismatch in which only donor cells respond [34]. Splenocytes were harvested after 3 d and incubated with CD4-PE, CD8-PE, CD25-PE, CD69-PE and biotin-conjugated anti-H2K^d^ and anti-H2D^d^ mAbs. Donor-specific alloreactive T cells were identified by gating on H-2K^d^- and H-2D^d^- cells (FACSCalibur; BD Biosciences), and T cell proliferation was assessed by CFSE division profiles [35].

For intracellular cytokine staining, splenocytes (3×10^6^/ml) were stimulated for 4 h with PMA (3 ng/ml) /ionomycin (1 µM) in 24-well plates in complete medium (RPMI 1640, 10% FCS, 2-ME, penicillin /streptomycin) in the presence of Golgi-Stop (BD-PharMingen). Cells were stained with cell surface markers (CD4-PE or CD8-PE, biotin-conjugated H-2K^d^ or H-2D^d^ mAbs followed by SA-Percp), fixed, permeabilized and with anti-IFN-γ-APC or anti-IL-2-APC mAb. Foxp3 expression was detected according to the manufacturer's instructions (eBioscience). Briefly, cells were labeled with surface markers and fixed overnight, permeabilized and stained with rat anti-Foxp3-PE mAb (rat IgG2a isotype control) and rabbit-anti-mouse Nurr77 (rabbit IgG isotype control), followed by FITC-conjugated anti-rabbit IgG, and analysis (FACSCalibur).

### T cell purification and stimulation

CD4+ T cell were negatively selected (>90%) with magnetic beads (Miltenyi) and separated into CD25+ and CD25− populations; purity was >90% as assessed by flow cytometry. T cells were stimulated in vitro with soluble or plate-bound CD3 mAb, CD3 plus CD28 mAbs (BD Pharmingen) or PMA (3 ng/ml)/ionomycin (1 µM) plus IL-2.

### qPCR

RNA was extracted using Rneasy® Mini Kit (QIAGEN) and reverse transcription of RNA samples (2 µg) performed with random hexamers (ABI PRISM 5700, Applied Biosystems). Specific primer and probe sequences for target genes (TaqMan PDAR, Applied Biosystems) were used for qPCR amplification of total cDNA (50 ng). Relative quantitation of target cDNA was determined by setting the control value to 1; changes in cDNA content were expressed as fold increases above the set control value. Differences in cDNA input were corrected by normalizing signals obtained with specific primers to ribosomal RNA. Nonspecific amplification was excluded by performing RT-PCR reactions without target cDNA.

### DBTC treatment in vitro and in vivo

DBTC was dissolved in ethanol absolute to make a stock solution. For *in vitro* experiments, cells were incubated with 3 µM DBTC dissolved in 0.1% ethanol (final concentrations). For *in vivo* treatment, DBTC stock solution was diluted in corn oil and given by gavage at a dose of 60 mg/kg; control animals received the same volume of corn oil and 5% ethanol.

### Annexin V staining

We performed qualitative cell apoptosis assay using the Annexin V-PE apoptosis detection kit (BD-Pharmingen) according to the manufacturer's instructions. Briefly, cells were washed twice with cold PBS, resuspended in binding buffer (1×10^6^ cells/ml), 100 µl of solution was incubated with 5 µl of Annexin V-PE and 5 µl of 7-AAD for 15 min at RT in the dark, and 400 µl of binding buffer was added and cells analyzed by flow cytometry.

### In vitro suppression assay

Assays of Treg function in vitro were performed using MACS-purified CD4+CD25− T cells as effector cells and CD4+CD25+ cells or retroviral transfected Foxp3 expressing CD4+ T cells as regulatory cells. The proliferation of CFSE-labeled CD4+CD25− cell, driven by CD3 mAb (0.5 µg/ml) plus γ-irradiated syngeneic APC, was assessed by flow cytometric analysis of CFSE dilution after 72 h [21].

### ELISPOT assay

Immunospot assays for IFN-γ were performed by coating ELISPOT plates (BD Pharmingen) with anti-IFN-γ mAb, followed by blocking, addition of MACS-purified CD3+ T cells isolated from cardiac transplant recipient spleens, and use of plus irradiated (2000 Rad) donor splenocytes as stimulators; recipient splenocytes served as syngeneic controls [21]. After 24 h, cells were discarded and wells washed, followed by use of biotinylated anti-IFN-γ mAb, streptavidin-HRP and substrate reaction. Spots were counted automatically using ImmunoSpot Analyzer (Cellular Technology). Recipient anti-donor responder frequency was determined by IFN-γ spot-forming cells (SFC)/2×10^5^ recipient splenocytes.

### Western blots

Grafts were sonicated in lysis buffer containing Triton X-100 and protease inhibitors, followed by centrifugation and quantification of supernatant protein content. Proteins were reduced, resolved by SDS-PAGE and transferred to nitrocellulose membranes. Membranes were blocked, incubated with primary and HRP-linked secondary antibodies, followed by substrate reaction and film development [21].

### Immunoprecipitation

Immunoprecipitation was performed using the Seize^®^ X Mammalian Immunoprecipitation Kit (PIERCE) according to the manufacturer's instructions. Briefly, antibodies were bound and cross-linked to immobilized protein G for 2 h in room temperature, 5×10^6^ cells were lysed using the M-PER® reagent, and extracted cell samples were mixed 1∶1 with Bind/Washing Buffer and added to the antibody-coupled gel in the spin cup column. Samples were incubated overnight at 4°C with end-over-end mixing, eluted with ImmunoPure^®^ elution buffer and prepared for SDS-PAGE [21].

### *In vivo* depletion of Treg and adoptive transfer

Tregs were depleted by undertaking thymectomy under tracheal intubation 7 d pre-transplant and daily injection of the depleting CD25 mAb, PC61 mAb (250 µg, i.p.), for 3 consecutive days post-thymectomy; the efficacy of depletion was monitored by flow cytometry [20]. For adoptive transfer, 2×10^6^ MACS-purified CD4+CD25− T cells from naive mice were injected i.v. into recipient mice immediately post-transplantation.

### Cardiac transplants

Intra-abdominal vascularized murine heart transplantation (BALB/c to C57BL/6) was performed by standard methods [36]. Briefly, donor ascending aorta and pulmonary artery were anastomosed end-to-side to recipient infrarenal aorta and inferior vena cava, respectively. Graft survival was assessed daily by abdominal palpation; rejection was defined as total cessation of cardiac contraction and was confirmed by histology. Portions of harvested allografts were fixed in formalin and paraffin embedded, or snap-frozen and analyzed by immunoperoxidase staining with a panel of mAbs.

### Statistics

Allograft survival was used to generate Kaplan-Meier survival curves, and comparison between groups was performed by log-rank analysis.

## Supporting Information

Figure S1 Pro- and anti-apoptotic gene expression by WT and Nur77Tg Tregs. CD4+CD25− and CD4+CD25+ cell population were isolated from WT and Nur77Tg mice using magnetic beads, followed by qPCR analysis (mean±SD) of gene expression in resting cells (black) or after 24 h of PMA/ionomycin stimulation (blue).
(0.80 MB PPT)

Figure S2 T cell apoptosis post cardiac allografting. Cardiac transplantation was performed from BALB/c donor to C57BL/6 or Nur77Tg recipients. Recipient (a) spleen and (b) lymph node cells were prepared 7 or 21 d later and stained with CD4, CD8 and Annexin V, followed by flow cytometry analysis. Data are expressed as dot plots, with the figure in each square indicating the percentage of Annexin V+ cells within the gated population. (c) Spleens and lymph nodes were harvested from WT or Nur77Tg recipients at 7 or 21 d after cardiac transplantation. Single cell suspension was prepared and stained with T cell markers and Foxp3. CD4+Foxp3+ T cell populations in the recipient secondary lymphoid organs were analyzed by flow cytometry. Data are expressed as dot plots, with the figure in each square indicating the percentage of positive cells within the gated population.
(4.85 MB PPT)
