# Supplementary figures and images for: Resistance of Foxp3+ Regulatory T Cells to Nur77-Induced Apoptosis Promotes Allograft Survival
Source: PLoS One. 2008 May 28;3(5):e2321. doi: 10.1371/journal.pone.0002321 (PMC2386419; doi:10.1371/journal.pone.0002321)

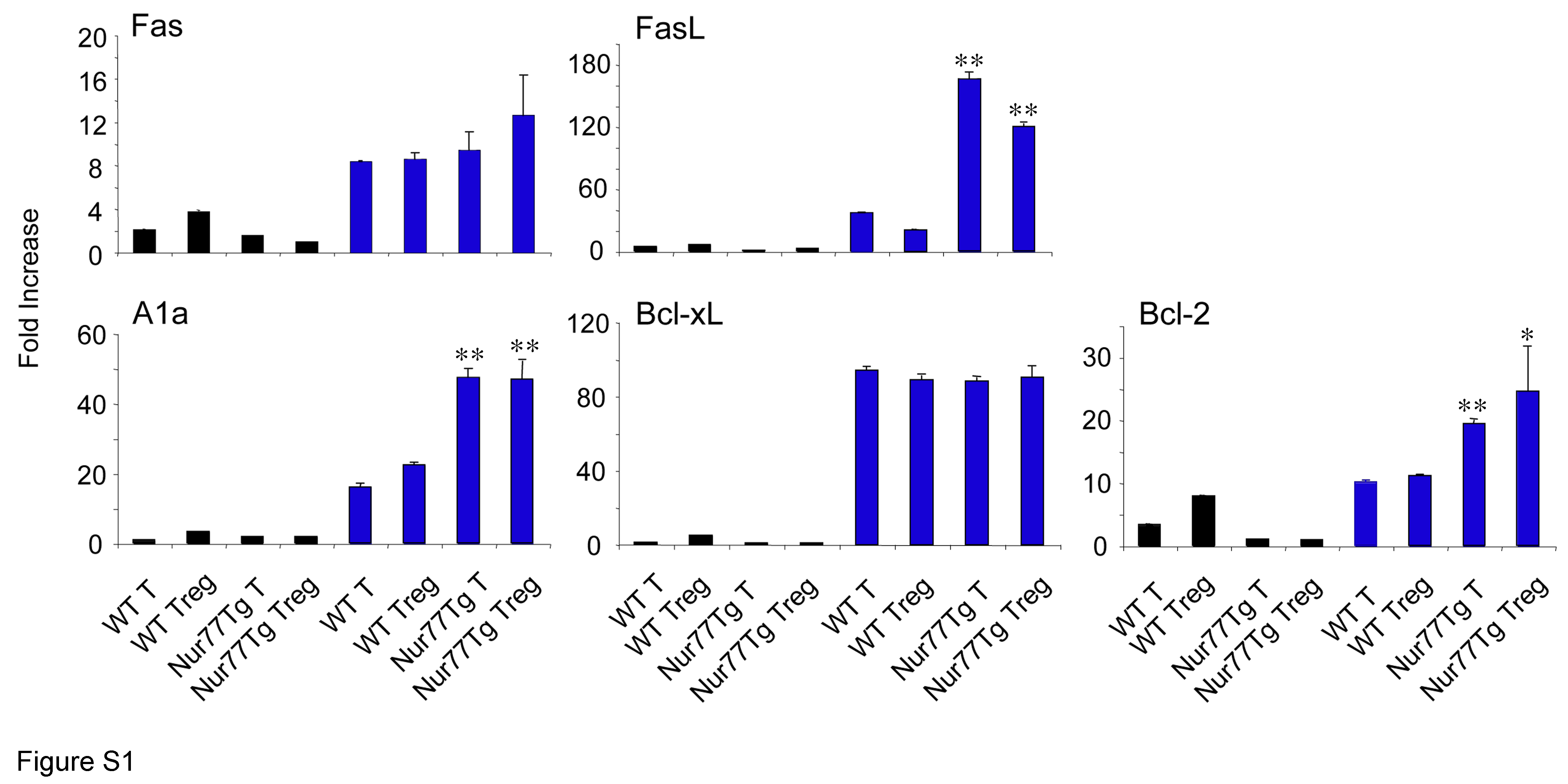

Supplement: Figure S1 — Pro- and anti-apoptotic gene expression by WT and Nur77Tg Tregs. CD4+CD25− and CD4+CD25+ cell population were isolated from WT and Nur77Tg mice using magnetic beads, followed by qPCR analysis (mean±SD) of gene expression in resting cells (black) or after 24 h of PMA/ionomycin stimulation (blue). (0.80 MB PPT) [file pone.0002321.s001.tif]

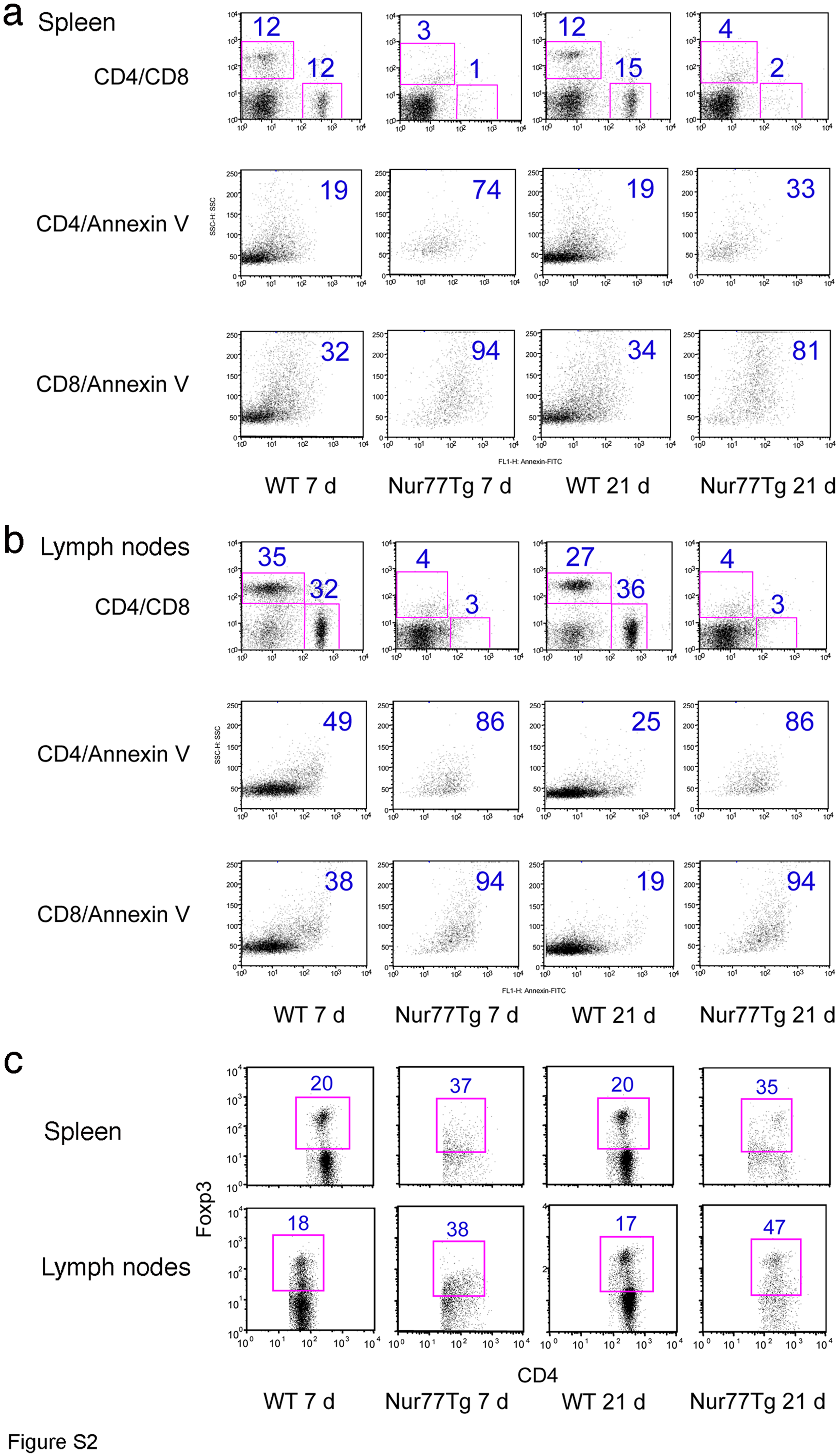

Supplement: Figure S2 — T cell apoptosis post cardiac allografting. Cardiac transplantation was performed from BALB/c donor to C57BL/6 or Nur77Tg recipients. Recipient (a) spleen and (b) lymph node cells were prepared 7 or 21 d later and stained with CD4, CD8 and Annexin V, followed by flow cytometry analysis. Data are expressed as dot plots, with the figure in each square indicating the percentage of Annexin V+ cells within the gated population. (c) Spleens and lymph nodes were harvested from WT or Nur77Tg recipients at 7 or 21 d after cardiac transplantation. Single cell suspension was prepared and stained with T cell markers and Foxp3. CD4+Foxp3+ T cell populations in the recipient secondary lymphoid organs were analyzed by flow cytometry. Data are expressed as dot plots, with the figure in each square indicating the percentage of positive cells within the gated population. (4.85 MB PPT) [file pone.0002321.s002.tif]
